# Enhancing Polymethyl Methacrylate Prostheses for Cranioplasty with Ti Mesh Inlays

**DOI:** 10.3390/jfb14080420

**Published:** 2023-08-10

**Authors:** Gargi Shankar Nayak, Heinz Palkowski, Adele Carradò

**Affiliations:** 1 Institute of Metallurgy (IMET), Clausthal University of Technology, Robert-Koch-Strasse 42, 38678 Clausthal-Zellerfeld, Germany; gargi.nayak@uni-saarland.de (G.S.N.); heinz.palkowski@tu-clausthal.de (H.P.); 2 Department of Applied Mechanics, Saarland University, Campus A4 2, 66123 Saarbruecken, Germany; 3 CNRS UMR 7504, Institut de Physique et Chimie des Matériaux de Strasbourg (IPCMS), Université de Strasbourg, 23 rue du Lœss BP 43, 67034 Strasbourg, France

**Keywords:** PMMA, Ti mesh, metal-mesh-reinforced composite, adapted mechanical properties for implants

## Abstract

Biocompatible polymers such as polymethyl methacrylate (PMMA), despite fulfilling biomedical aspects, lack the mechanical strength needed for hard-tissue implant applications. This gap can be closed by using composites with metallic reinforcements, as their adaptable mechanical properties can overcome this problem. Keeping this in mind, novel Ti-mesh-reinforced PMMA composites were developed. The influence of the orientation and volume fraction of the mesh on the mechanical properties of the composites was investigated. The composites were prepared by adding Ti meshes between PMMA layers, cured by hot-pressing above the glass transition temperature of PMMA, where the interdiffusion of PMMA through the spaces in the Ti mesh provided sufficient mechanical clamping and adhesion between the layers. The increase in the volume fraction of Ti led to a tremendous improvement in the mechanical properties of the composites. A significant anisotropic behaviour was analysed depending on the direction of the mesh. Furthermore, the shaping possibilities of these composites were investigated via four-point bending tests. High shaping possibility was found for these composites when they were shaped at elevated temperature. These promising results show the potential of these materials to be used for patient-specific implant applications.

## 1. Introduction

The implant industry has seen tremendous growth in recent years. To repair bone fractures, several innovative implants have been developed. However, the complexity of the human body always makes it difficult for any implant to perform as well as the native body part. The inertness of metallic implants such as Titanium (Ti) [1,2,3,4] makes it easy for the body to accept these implants, and their high mechanical strength provides sufficient stability at the fracture site, which promotes the bone healing process [5,6]. However, the significant difference in stiffness between the implant and the bone causes bone resorption over time, as the majority of stress is taken by the implant instead of the bone [7,8]. This phenomenon is known as “*stress shielding*”. Ceramics, on the other hand, have an excellent biocompatibility, but their brittleness, low toughness, and poor workability restrict their applications for load-bearing sites [9,10]. Polymers also have substantial advantages in terms of biocompatibility and processability, but their lack of mechanical strength and sterilisation difficulties make them limited to use for hard tissue replacements [11,12].

To mitigate these disadvantages, composite biomaterials were developed. These materials were created by combining various classes of biomaterials in a manner that minimises the limitations of their constituents while augmenting [13,14,15,16,17]. To reduce the disadvantages of metals, metal–polymer–metal sandwich materials were developed that not only reduced the stress shielding problem but also enhanced the strength-to-weight ratio and the energy absorption capacity [18]. Similarly, for improving the toughness of the ceramics, polymer fibres can be added [19]. 

In a similar fashion, the properties of the polymers can also be enhanced via reinforcements. In the past, reinforcements such as ceramic particles, metal particles, glass fibre (GF), carbon fibre (CF), and metal mesh were used for improving the mechanical properties of composite materials [20,21,22,23,24]. These composites are alluring for various fields of application due to their ease of production and significant mechanical performance improvement. These composites can be easily formed even without chemical bonding by the application of fusion bonding [25,26]. Fusion bonding occurs in two steps: (1) intimate contact and (2) the autohesion process [27,28]. 

Once the polymers are pressed together under temperatures above their glass transition temperature (*T_g_*), they come into close contact and the polymer chains start to diffuse across the interfaces due to Brownian motions [29,30]. This technique has been successfully used in the past to create thermoplastic composites [31,32].

Numerous studies have been conducted to examine the advantages of reinforced composites. Liao et al. investigated the influence of the addition of short carbon fibres (CF) (15–20 mm) on Polyamide (PA)-12 [33]. They added 2, 4, 6, 8, and 10 wt.% of CF on PA-12 melt and prepared the composites via melt compounding. As a result, at 10 wt.%, the CF enhanced the tensile strength of the composite by 102.2% and the flexural strength by 251.1%. On the other hand, the addition of anisotropic materials such as long CF or glass fibres (GF) or metal meshes resulted in orientation-dependent mechanical property enhancements of the reinforced polymers [34,35]. Moreover, Ali et al. showed that the addition of ceramic particles can lead to even further improvements in the mechanical properties of CF-reinforced composites [24]. In another study, Zhou et al. investigated the influence of the GF direction on the tensile properties of GF-reinforced PA-6.6 [36]. They stated that the tensile strength and elastic modulus (E) decreased to 35 and 43%, respectively, when the direction of the GF changed from parallel to perpendicular to the applied force direction. As bones are also anisotropic by nature [37], the addition of such materials can help in developing composite materials that can mimic bone behaviour to a certain extent. 

However, the problem with fibre-reinforced polymers is the lack of ductility of the CF and GF, which can make shaping hard for patient-specific implants (PSIs) [38]. This is not the case for metal reinforcements with their higher ductility. Due to this reason, metallic reinforcements have been used in the past to improve the ductility of polymers [34,39]. Hamidi et al. showed the benefits of the addition of metal wires to the mechanical properties of the polymers [22]. They used 3D printing (fused deposition modelling) to reinforce polylactic acid (PLA) with steel wire and copper wire. While copper did not show a notable effect on the tensile strength, steel wire addition led to a 60% increase in the tensile strength. In another study, Ibrahim et al. improved the impact strength of polymethyl methacrylate (PMMA) via the application of stainless steel 316L (SS316L) for denture applications [40]. 

The aim of this study was to apply a metallic mesh that can enhance the properties of polymeric materials for cranioplastic applications. PMMA was used as the matrix, and Ti mesh was used as reinforcement, as both of these materials are biocompatible by nature. This idea was based on the fact that PMMA is a widely used material for prosthetic applications but lacks the mechanical properties to be used as an implant where load can be applied [41,42]. This enhancement can improve the potential of PMMA for further implant applications. The ductility of the metallic mesh can even help in improving the shapability of this composite. Hot-pressing was used to assemble the composites to achieve adhesion via fusion bonding between the polymers. The influence of vol.% (number of mesh layers) and direction of the Ti meshes on the mechanical properties of the reinforced composites was studied. Moreover, as these composites need to be shaped to be available as implants for patient-specific implant (PSI) applications, their shapability was also studied via four-point bending tests after determining the necessary conditions for shaping PMMA. The bending test is easy to perform and gives basic information about failure by cracking and/or delamination. 

## 2. Materials and Methods

**Figure 1:**
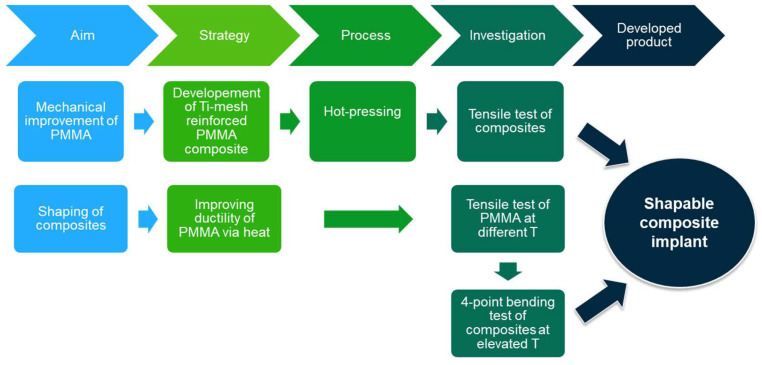
Investigation methodology applied in the study.

The investigation methodology applied in this study is sketched in Figure 1.

### 2.1. Materials Used

**Figure 2:**
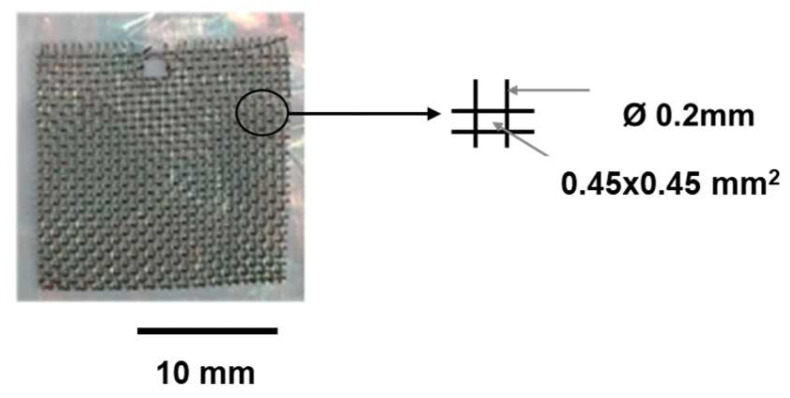
Geometry of the used Ti mesh.

Plexiglass PMMA 0F301 (0.3 mm thick, Evonik Industries AG, Wesseling, Germany) and Ti grade 2 mesh (Baoji Hanz Material Co. Ltd., Shaanxi, China) were used as initial materials in this study. The mesh dimensions are given in Figure 2.

**Figure 3:**
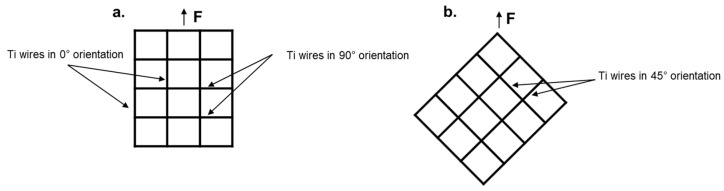
Orientation representation of Ti meshes. (**a**). Ti mesh in 0°/90° orientation; (**b**). Ti mesh in 45° orientation.

For investigating the influence of the Ti mesh vol% on the mechanical properties of the final composites, 1, 2, 3, and 4 layers of the Ti mesh were placed between PMMA foils in 45° orientation. Moreover, to understand the influence of mesh orientation on the mechanical properties of the composites, the tensile properties of 4-layered Ti-mesh-reinforced PMMA with 0°/90° orientation were used for comparison (see Figure 3). The abbreviations and details of the specimens used in this study are shown in Table 1.

### 2.2. Sample Preparation

**Figure 4:**
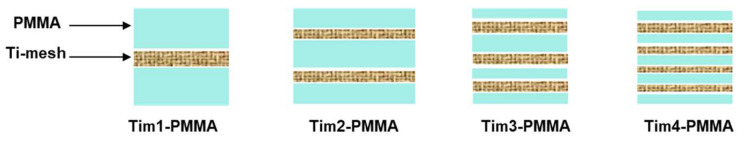
Ti mesh and PMMA layer arrangement for preparation of the composites. For a constant total thickness of 1.5 mm, the thickness of PMMA was adapted accordingly.

A Ti mesh of dimension of 200 × 200 mm^2^ was initially taken and rinsed using an ethanol solution in an ultrasonication bath for 5 min to remove impurities. No additional preparation was carried out. PMMA foils of similar dimensions were also taken and cleaned using an ethanol solution. The PMMA foils and Ti meshes were stacked together based on the type of specimen to be developed and finally wrapped together using a Teflon foil to avoid any lateral motion during the hot pressing. This also prevented the PMMA foils from getting glued to the hot-press surface during processing. Hot pressing, with the parameters T = 150 °C, t = 150 min, and *p* = 2 bar, was performed afterwards. The parameters were taken based on previous studies [43]. The thickness of all the specimens was kept constant with 1.5 mm for comparison. This led to differences in the distance between the mesh layers in the different composites. Whereas for 4 layers of mesh, the distance between each layer was uniform and close to each other, for 3 layers, the distance between the first and second layers was greater than that between the second and third layers. The arrangement of the layers for the composites can be seen in Figure 4.

### 2.3. Performed Tests

Tensile tests: Tensile testing was performed following DIN EN ISO 527-5-A2; a universal testing machine (UTS 250 kN) was used to test the PMMA and composites for uniaxial tensile strength. The standard tests were performed at room temperature (RT) with a test speed of 0.1 mm/min. 

### 2.4. Shaping at Elevated Temperatures

Tensile tests of PMMA at different temperatures: As PMMA is brittle at room temperature, the shaping of these composites needs a temperature regime where the polymer has a sufficient ductility without becoming viscous, because this has a negative effect on shaping [44]. Thus, to evaluate this temperature range, uniaxial tensile tests on PMMA were performed following DIN EN ISO 527-3-1B at temperatures of 20, 40, 60, and 80 °C with a constant strain rate of 0.001 s^−1^ using the above-mentioned universal testing machine UTS.

Bending tests: To obtain a first and basic idea of the shapability of these composites, 4-point bending tests were performed following DIN EN ISO 14125. The specimens with dimensions 80 × 15 × 1.5 mm^3^ were prepared using hot pressing under the same conditions as mentioned before. Subsequently, the tests for all the specimens were performed at RT. A punch velocity of 1 mm/s was used for the bending tests. Afterwards, following the results obtained concerning the temperature dependent ductility of PMMA, 4-point bending tests were performed for all the composites at a temperature of 80 °C to compare the influence of temperature—and therewith ductility of the polymer—on the formability of these structures. 

## 3. Results and Discussion

### 3.1. Cross-Section Analysis 

**Figure 5:**
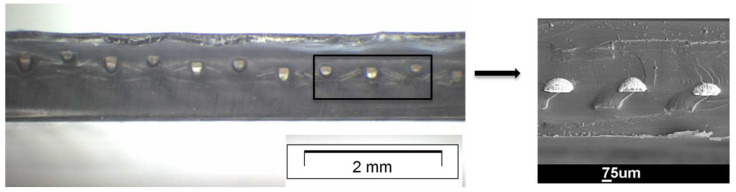
Cross-section analysis via LOM (left) and SEM (right) of prepared Tim1-PMMA specimen. No delamination could be detected.

The cross-sections of the composite specimens were analysed using light-optical microscopy (LOM) and scanning electron microscopy (SEM) analysis. An example is given in Figure 5, showing that hot pressing could in general be stated to be successful in achieving good fusion bonding between a Ti mesh and PMMA. The white balls represent the ends of the cut Ti wires surrounded by the polymer (dark).

### 3.2. Tensile Test

**Figure 6:**
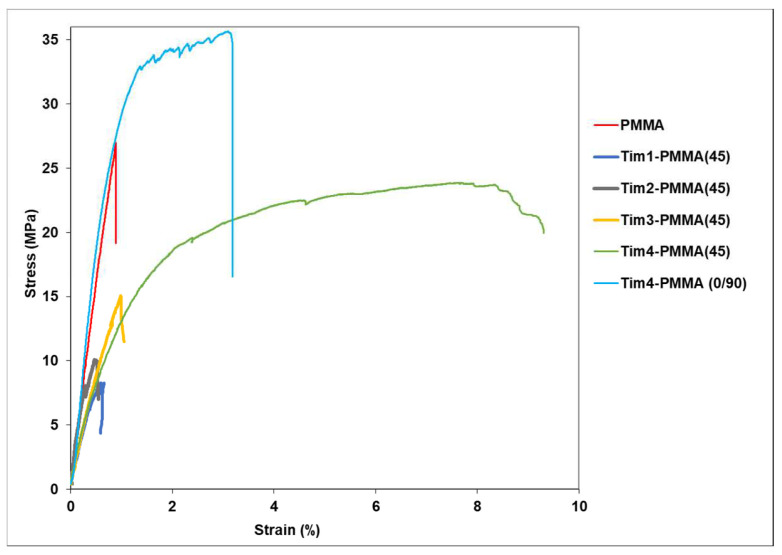
Tensile test results of all types of the specimens. A significant improvement in Tim4-PMMA in 0, 90° orientation improved the strength because of the stiffening effect of the Ti wires in the longitudinal direction; with 45° orientation, shearing was dominant, improving the ductility at the expense of strength.

The tensile test results obtained at room temperature for all the specimens are shown as an example in Figure 6. 

**Figure 7:**
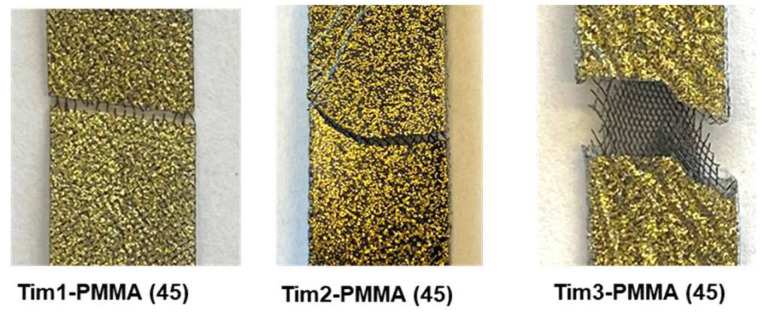
Fractured Tim-PMMA specimens.

**Figure 8:**
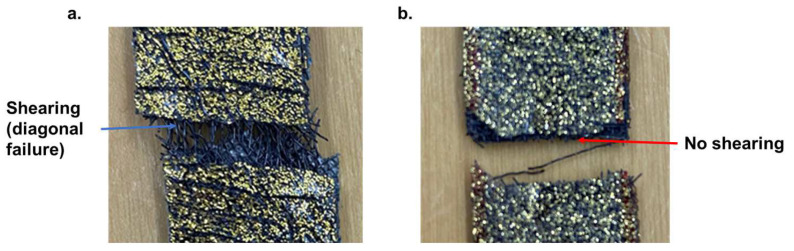
Fracture after tensile test: (**a**). Tim4-PMMA (45), (**b**). Tim4-PMMA (0°/90°). A shearing effect was observed for a 45° orientation of the Ti mesh but not for a 0°/90° orientation.

The initial result of the addition of Ti meshes showed a significant decrease in tensile strength in comparison to pure PMMA. Up until the addition of three layers of Ti mesh, the specimens showed early failure; see Figure 7. However, a clear improvement in ductility was observed when four layers of Ti mesh were placed in a 45° orientation. Respectively, the strength rose strongly when these amounts of Ti mesh were placed in 0° and 90° orientations (Table 2). Moreover, the shearing effect was predominant for the 45° orientation of the Ti mesh (fracture in diagonal direction), which was not the case for the 90° orientation (see Figure 8).

**Figure 9:**
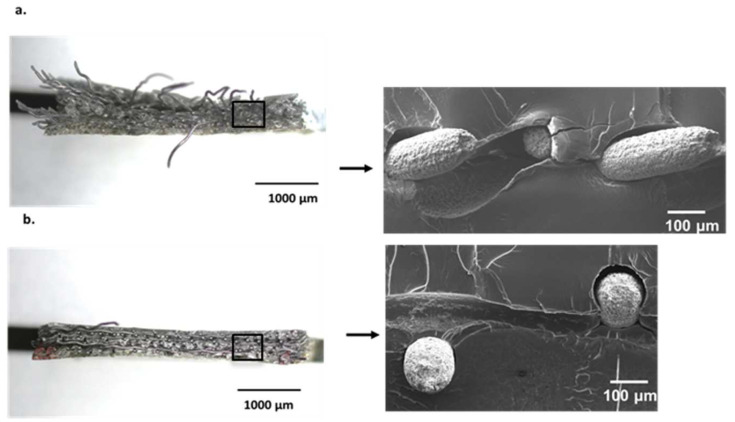
(**a**). LOM (**left**) and SEM (**right**) images of Tim4-PMMA (45) displaying extreme deformation of the Ti mesh; (**b**). LOM and SEM (**right**) images; image of Tim4-PMMA (0°/90°) (**left**), exhibiting sample failure along the same axis for the Ti mesh without high deformation.

**Figure 10:**
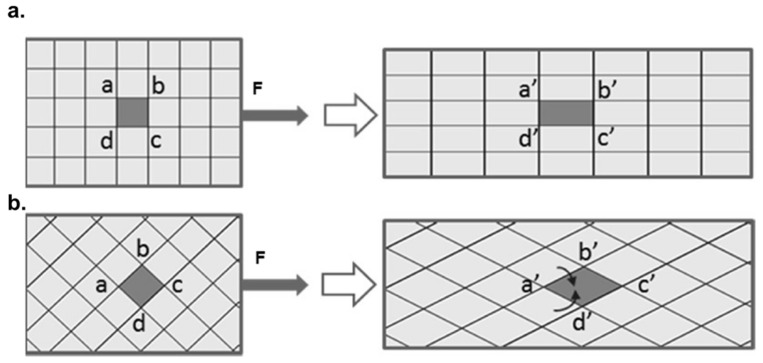
Schematic of the influence of orientation on the elongation of mesh under load. (**a**). 0°/90° orientation; (**b**). 45° orientation.

In LOM and SEM analysis, the fractured surface for a 45° orientation showed a high deformation of the Ti mesh, whereas it failed uniformly at one point for 0°/90° orientation (Figure 9). The deformation and load-bearing capacity of the Ti mesh, and therewith the mesh-containing composites, are highly dependent on their load paths; see Figure 10. When the orientation of the mesh was 0°/90°, the deformation led to a rectangular shape change where the elongation of only the mesh in 0° orientation supported the applied load. On the other hand, when the orientation of the Ti mesh was 45°, the load-initiated deformation led, as expected, to a rhombohedral shape change, where all the sides of the rhombohedral became elongated in the tensile direction. Thus, the load was mainly taken by the polymer with an increased deformation for Tim4-PMMA in the 45° orientation in comparison to the 0°/90° one. This is the reason behind the higher elongation of Tim4-PMMA in the 45° direction. This mechanism is also responsible for the shear elongation of Tim4-PMMA [45]. 

Another important aspect is the stiffness, which depends on the anisotropy of the mesh. For the same volume fraction of the Ti mesh, the Young’s modulus of the composite can be increased or decreased. Where the E-value of PMMA was found to be 3.1 GPa, it decreased clearly to 2.2 GPa for the Tim4-PMMA (45) and increased to 4.8 GPa for the Tim4-PMMA (0°/90°). This is due to the fact that the applied forces act on the Ti mesh differently. The wires that can take part in carrying the load are dependent on their orientation. The part of the applied force acting on the Ti wires is only Fcosθ, where θ is the angle between the Ti-wire orientation in the mesh and the applied force direction. Due to this reason, at a 0°/90° orientation of the Ti mesh, the Ti wires in the force direction (0° orientation) can take the maximum force (cos 0° = 1), thereby improving the stiffness of the composite clearly. Under these conditions, more force is taken by the mesh for elongation, thus increasing the Young’s modulus of the composite. On the other hand, for a 45° orientation, less load can be carried (Fcos45°), which is approximately 0.7 times the applied load. This, along with the fact that the elastic elongation is higher in the case of a 45° orientation of the mesh, decreased the E-value of the composite [20]. The localised stress induced by the shear elongation of the mesh in this case led to the formation of shear bands in the PMMA, thus maximising its plastic deformation [46]. Similar results have been obtained in other studies [47,48]. In the study performed by A. Parmiggiani et al., the influence of the orientation of the carbon fibres on the mechanical properties of CF-reinforced thermoplastics was investigated [49]. Where for the 0° orientation of the CF the tensile strength seemed to increase clearly, for the 45° orientation a strong increase in ductility was found.

The reason for the poor performance of Tim1, Tim2, and Tim3-PMMA (45) could be an uneven load distribution between the Ti mesh and the polymer, along with the low volume fraction of the Ti mesh. For the Tim1-PMMA, the volume fraction of the Ti mesh in the PMMA is too low to produce a clear effect. However, for Tim2 and Tim3-PMMA, the distance between Ti mesh layers is too large, which inhibits their influence on the overall performance of the composites. It also needs to be considered that PMMA is brittle at room temperature, so in regions where bulk PMMA is in high quantity, craze propagation can initiate. Thus, if the distance between the mesh layers is too large, the crack propagation of PMMA cannot be hindered by the mesh, minimising the reinforcement effect as a result. As with higher vol% of the mesh, not only will the load be better carried by the meshes, which can minimise cracks in the PMMA, but fracture propagation will also be hindered, as was stated in [50,51].

The maximum deformation was found to be higher for both 0°/90° and 45° orientations of Tim4-PMMA, compared to that of pure PMMA. The shear band effect of the reinforcement could be one reason behind this behaviour [52]. The high stress concentration around the periphery of the reinforcement leads to localised shear band formation. As the ductility of polymers increases with higher shear band formation [53], the max. strain before fracture increases for the Tim4-PMMA, which was even higher for the 45° orientation due to the higher shearing of the Ti mesh under these loading conditions. 

### 3.3. Tensile Tests of PMMA at Different Temperatures

**Figure 11:**
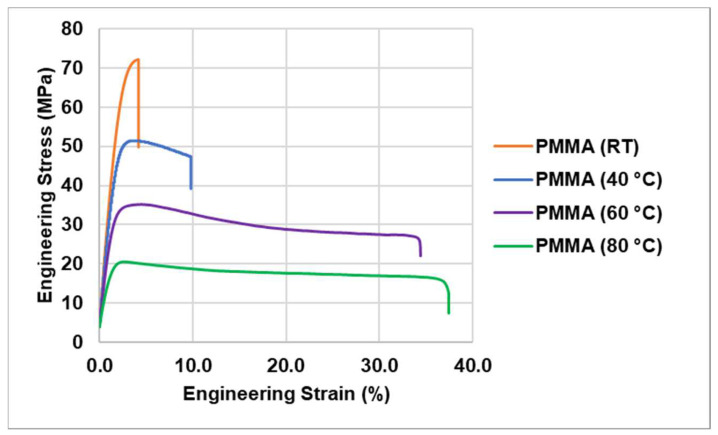
Tensile test results of PMMA at different temperatures. The ductility increases strongly with increasing T; the strength decreases analogously.

As compared to room temperature, a considerable improvement in the ductility of PMMA was found at elevated temperatures (see Figure 11). Not only did the maximum strain increase, but the shaping became feasible at lower engineering stresses. 

This occurred due to the brittle-to-ductile transition of PMMA with increasing temperature [44,54]. Generally, in PMMA the deformation can lead to crazing or shear band formation. Where crazing leads to void formation, causing brittle failure, shear band formation improves the ductility by suppressing new craze initiation and propagation of existing crazes. The majority of crazing or shear band formation determines the ductility of the PMMA. At lower temperatures, the activation energy for crazing is lower than the one of shear band formation which leads to early failure. However, this changes with the increase in temperature, as the activation energy required for shear band formation gets lower, consequently significantly improving the ductility of the PMMA [55]. 

Another important reason behind this mechanism is the β-relaxation of PMMA. At temperatures T < *T_g_*, the β-relaxation is the major relaxation that affects the mechanical properties of PMMA [56]. At a low temperature, the entanglements between secondary bonds are strong enough to withhold their structure until failure. However, with increasing T, these secondary bonds start to fail at lower loads due to the β-relaxation, which increases the ductility of the PMMA by a weak entanglement effect. It starts around 40 °C for PMMA, thus improving its ductility moving forward [57]. 

### 3.4. Bending Tests 

**Figure 12:**
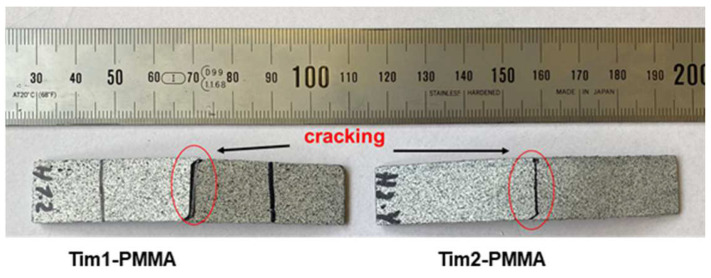
A crack occurred during four-point bending of Tim1- and Tim2-PMMA (45) specimens.

**Figure 13:**
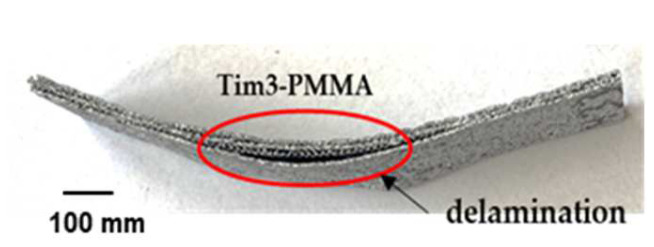
Tim3-PMMA specimen after four-point bending. The specimen suffered delamination during the process.

**Figure 14:**
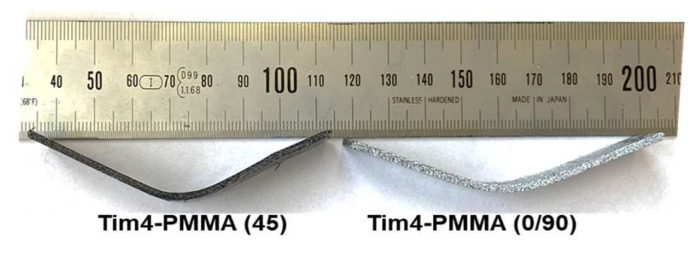
Tim4-PMMA (45) and Tim4-PMMA (0/90) specimens after bending up to 90°. A springback effect of approx. 30° was seen in both specimens.

The visual bending results at failure for Tim1- and Tim2-PMMA (45) specimens at RT are shown in Figure 12. Delamination occurred for the Tim3-PMMA (45) specimens; see Figure 13, not to be seen in Figure 12 Tim4-PMMA specimens for both orientations. A springback factor (bending angle/bent angle) of 3 was found for both specimens, showing a strong elastic behaviour of the Tim4-PMMA component, as shown in Figure 14.

**Figure 15:**
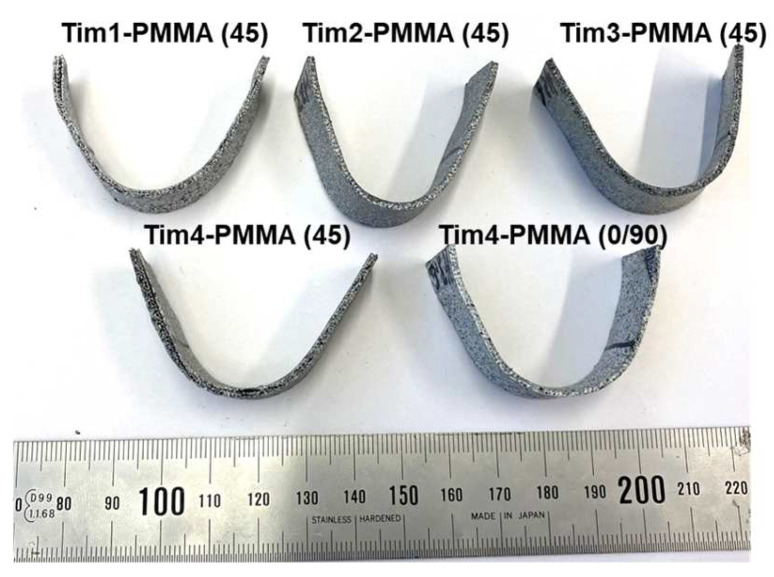
Fully bent all Tim-PMMA composites after four-point bending at 80 °C and subsequent water cooling.

However, when bending was performed at 80 °C, where PMMA was found to have sufficient ductility, all the specimens were able to be shaped without failure. After reaching the final bending position, the specimens were instantly taken out of the chamber and subsequently quenched in water to retain their final shape; see Figure 15. The springback factor was found to be 1 in this case for all the specimens.

The bending results show the importance of Ti mesh in the load-bearing capacity of Tim-PMMA composites. For only one and two layers of Ti mesh, the load is mostly carried by the PMMA, due to the lower volume fraction of the mesh in the composites, resulting in an early failure of the outer layer under tension conditions. However, this changed for the three- and four-layer conditions of Tim-PMMA composites. Here, the composites were able to be bent to the full extent at RT without failure, where the PMMA is quite brittle. However, the localised plastic deformation in the periphery of the Ti mesh needed to be shared uniformly by all the layers, which was not the case for Tim3-PMMA, where the Ti meshes were not placed uniformly in the loading direction (the distance between Ti mesh layers is not homogeneous, so to keep the thickness of the composite the same in all the cases, a thicker PMMA layer was placed between the first and second layer of the Ti mesh compared to the second and third one). Thus, the shearing effect of the Ti mesh led to earlier delamination [58]. This was not the case for Tim4-PMMA specimens, where the Ti meshes were evenly distributed within the composite and could carry the load successfully. Thus, no delamination was found for the latter specimen. The strong elastic recovery found for Tim4-PMMA at RT could be due to the fact that the bending of the Ti mesh inside the composite was elastic; thus, once the load was removed, they preferred to achieve the initial position once again. The remaining small plastic deformation could be due to the shear-band-induced deformation of the PMMA [52]. The results can be correlated to the results gained by B. Magyar et al. where they improved the flexural performance of PCL via carbon fibre reinforcements [38]. They reported that the increased energy absorption capacity at the polymer-CF interface was expected to be responsible for this behaviour.

The bending of composites at an elevated temperature of 80 °C, where the PMMA is sufficiently ductile, was successful considering non-failure in all cases of the different layer combinations. At this temperature, PMMA has sufficient ductility to allow shaping without becoming viscous which can lead to the failure of the composites. However, achieving the final shape under bending conditions is only possible for the Ti-mesh-reinforced PMMA if the final shape is frozen, i.e., if the PMMA is cooled down under load conditions, thus hindering the springback effect induced by the Ti meshes. Once cooled down, PMMA stiffens; thus, the shape of Ti-mesh-reinforced PMMA will not restructure unless heat is provided again. 

## 4. Conclusions

In this study, the influence of Ti meshes inlaid in a PMMA-matrix on enhancing the mechanical properties was investigated. The mechanical properties of these composites were initially investigated via tensile tests. The presence of meshes enhanced the mechanical behaviour strongly and clearly, but only if the volume fraction of the mesh was sufficient to carry the load during deformation and if the layering fit. When the ratio of the meshes was not high enough, they harmed the mechanical properties of the composite by reducing its ductility. For a composite with four inserted meshes, Tim4-PMMA, the mechanical properties improved significantly, where the properties of the final composite were dependent on the orientation of the mesh to the load direction. In 45° orientation, they helped in improving the ductility as well as slightly increasing the strength because of the shearing tendency of the mesh, whereas for 0°/90° orientation they clearly strengthened the composite along with a minor improvement in ductility. 

To facilitate the shaping of these composites, the ductility of the PMMA needs to be improved to overcome its brittleness at room temperature. With increasing temperature, the ductility of PMMA improved, and finally 80 °C was selected as the fitting shaping temperature for further studies.

Finally, the bending studies showed an enhancement by the mesh in the bendability of these composites. With a higher volume fraction, the composites were able to bend successfully in four-point bending tests up to 90°. During bending at RT, the meshes were mainly elastically deformed as the bending only induced shear and slide motion. This resulted in a strong springback effect from the Tim4-PMMA. However, this was not the case for bending at elevated temperatures (80 °C), where the PMMA was soft and more ductile. At this temperature range, all the mesh-reinforced PMMA composites could be shaped without failure, given they were cooled to that state to stiffen the PMMA before unloading. 

The success of this research opens the possibility of applying such metal-mesh-reinforced polymers in the field of PSI applications. These composites also have the advantage of adding functional qualities via surface modification of the polymers. In the next stages, this possibility will be investigated, along with applying this technique to other metal/polymer combinations. Moreover, to secure even stronger bonding in these composites, the metal mesh can be coated with polymers via the “grafting from” technique, thus being directly bonded with the bulk polymers via fusion bonding. 

## Figures and Tables

**Table 1:** Types and details of Ti-mesh (named here Tim)-reinforced PMMA.

Specimen Abbreviation	Ti Mesh vol.%	No. of Layer(s) Ti Mesh	Mesh Orientation (°)
Tim1-PMMA (45)	6	1	45
Tim2-PMMA (45)	12	2	45
Tim3-PMMA (45)	18	3	45
Tim4-PMMA (45)	24	4	45
Tim4-PMMA (0/90)	24	4	0/90

**Table 2:** Influence of Ti-mesh orientation on the mechanical properties.

Material/ Combination	Thickness (mm)	E (GPa)	UTS (MPa)	ER (%)
PMMA	1.5	3.1 ± 0.2	26.7 ± 5.0	0.8
Tim4-PMMA (0/90°)	1.5	4.8 ± 0.3	37.5 ± 6.0	3.4
Tim4-PMMA (45)	1.5	2.5 ± 0.2	21.5 ± 3.0	11

## Data Availability

The data presented in this study can be obtained on request from the corresponding author.
